# Apathy is associated with executive functioning in first episode psychosis

**DOI:** 10.1186/1471-244X-9-1

**Published:** 2009-01-08

**Authors:** Ann Faerden, Anja Vaskinn, Arnstein Finset, Ingrid Agartz, Elizabeth Ann Barrett, Svein Friis, Carmen Simonsen, Ole A Andreassen, Ingrid Melle

**Affiliations:** 1 Ullevål University Hospital, 0407 Oslo, Norway; 2 Aker University Hospital, 0320 Oslo, Norway; 3 Institute of behavioral sciences in medicine, University of Oslo, 0317 Oslo, Norway; 4 Diakonhjemmet Hospital, 0319 Oslo, Norway; 5 Institute of Psychiatry, University of Oslo, 0318 Oslo, Norway; 6 Institute of Psychology, University of Oslo, 0317 Oslo, Norway

## Abstract

**Background:**

The underlying nature of negative symptoms in psychosis is poorly understood. Investigation of the relationship between the different negative subsymptoms and neurocognition is one approach to understand more of the underlying nature. Apathy, one of the subsymptoms, is also a common symptom in other brain disorders. Its association with neurocognition, in particular executive functioning, is well documented in other brain disorders, but only studied in one former study of chronic patients with schizophrenia. This study investigates the association between apathy and neurocognitive functioning in patients with first episode psychosis (FEP), with the hypothesis that apathy is more associated with tests representing executive function than tests representing other neurocognitive domains.

**Methods:**

Seventy-one FEP patients were assessed with an extensive neuropsychological test battery. Level of apathy was assessed with the abridged Apathy Evaluation Scale (AES-C-Apathy).

**Results:**

AES-C-Apathy was only significantly associated with tests from the executive domain [Semantic fluency (r = .37, p < .01), Phonetic fluency (r = .25, p < .05)] and working memory [Letter Number Span (r = .26; p =< .05)]; the first two representing the initiation part of executive function. Confounding variables such as co-occuring depression, positive symptoms or use of antipsychotic medication did not significantly influence the results.

**Conclusion:**

We replicated in FEP patients the relationship between apathy and executive functioning reported in another study for chronic patients with schizophrenia. We also found apathy in FEP to have the same relationship to executive functioning, as assessed with the Verbal fluency tests, as that reported in patients with other brain disorders, pointing to a common underlying nature of this symptom across disorders.

## Background

Negative symptoms are common in patients with psychosis, but the underlying mechanisms are still poorly understood [1]. Neurocognitive deficits are also common in schizophrenia spectrum disorders, and are thought to be more closely linked to the biological underpinnings of the disorder than clinical symptoms [2]. As negative symptoms is repeatedly shown to have a consistent relationship with neurocognitive deficits [3-6], a further exploration of their relationship may aid the search for the mechanisms behind negative symptoms. A recent review by Harvey et al suggested four models as potential explanations for their association [7]: 1) the two categories of symptoms represent the same identical features or alternate manifestations of the same basic underlying process, or 2) the two features of the illness are separable but share similar underlying etiological factors, or 3) the two are of separate, but related etiologies, or 4) the two dimensions are distinct from each other and with separate etiology. The review concluded by inviting innovations in the assessment of negative symptoms to come closer to an understanding of negative symptoms and other aspects of psychosis [7].

One problem in studies of negative symptoms is the heterogeneity of patient samples examined, with an admixture of chronic samples marked by treatment failures, institutionalization, hopelessness and subsequent social consequences that are difficult to distinguish from primary negative symptoms based solely on behavioral observations. Another problem is the diverse nature of the negative symptoms per se, as it includes several subsymptoms (apathy or avolition, anhedonia, alogia, asociality, flat affect and inattention) that might have different etiologies. Based on the recognition of this diversity, the "NIMH-MATRICS consensus statement on negative symptoms" has suggested that studies specific to the nature of these negative subsymptoms is one way to move forward [1]. So far, very few studies on the association between negative symptoms and neurocognitive function have included analysis on the subsymptom level. We have thus found only four studies addressing the specific relationship between any of the negative subsymptoms and neurocognitive function [8-11]; the subsymptoms studied being alogia [8,10] flat affect [8,11] and apathy [9].

Apathy, defined as lack of motivation or goal directed behavior [12], has lately been targeted as an important negative subsymptom that need further study [13-15]. However, the only study concerned with the association between apathy and neurocognition in psychosis is based on a small sample of chronic patients with schizophrenia and a narrow test battery [9]. Here, a high level of apathy was associated with lower performance IQ scores and poor performance on tests assessing executive function and visual- and verbal memory. The authors called for new studies in order to generalize their findings, due to the small sample size and lack of a comprehensive neuropsychological test battery. Studying patients with their first episode of psychosis (FEP) is of particular interest, since the negative symptoms in such a sample cannot be secondary to chronic effects, treatment failures or social deprivation.

Apathy is considered a symptom arising from the prefrontal cortex [16] or from dysfunction in the frontal-subcortical circuits [17]. It's relationship to neurocognitive function has been extensively studied in other brain disorders, such as Alzheimer – (AD) [18], Parkinson – (PD) [19] and Huntington disease (HD) [20] in addition to traumatic brain injury (TBI) [21]. Common to studies of all the above disorders is the finding of a consistent relationship between high levels of apathy and poorer performance on tests representing executive function [19-24]. In addition, significant associations are found to other neurocognitive domains, especially working memory [19,24], psychomotor speed [21], attention [20] and episodic memory [20,21], but for these domains the pattern of association is less systematic.

The aim of the present study is to improve our understanding of apathy in FEP patients. Consequently, we wish to investigate the association between apathy and neurocognitive function. First, we hypothesize that also in FEP patients the degree of apathy will be significantly related to tests representing executive function, and less with tests representing other neurocognitive domains. Secondly, we hypothesize that this relationship is not influenced by confounding variables, such as depression, use of antipsychotic medication or positive symptoms, supporting the notion of a linked etiology between the two areas.

## Methods

### 2.1 Participants

The present study includes 71 FEP patients with a fluent understanding of Norwegian, recruited between July 2004 and end of June 2006. They all took part in both the clinical and the neuropsychological assessment in the ongoing Thematically Organized Psychosis (TOP) research study in Oslo, Norway. All patients were assessed in a stable phase. The neurocognitive testing was done in as close connection to the clinical assessment as possible, within one to eight weeks. Inclusion criteria were: Age between 18 and 65 years, with a first episode of psychosis and a DSM-IV diagnosis of either schizophrenia, schizophreniform disorder, schizoaffective disorder (constituting schizophrenia spectrum disorders); psychosis NOS, delusional disorder, brief psychosis (constituting other psychotic disorders), or affective disorder with mood incongruent psychotic symptoms and bipolar disorder (constituting affective psychotic disorders). Patients were eligible for inclusion up to 52 weeks following the start of the first adequate treatment. Being psychotic was defined as having a rating of 4 or more on anyone of the PANSS items P1, P3, P5, P6 or G9. Fifty seven (80.3%) of all used antipsychotic medication (AP); 51 on monotheraphy and six on two AP's. Of these 57, three were using first generation AP [Zuclopentixol (N = 2); Perphenazine (N = 1)] and the rest second generation AP [Olanzapine (N = 28); Risperidone (N = 9); Ziprazidone (N = 9); Quetiapine (N = 7), Aripriprazole (N = 5) and Amisulpride (N 01)]. The average Defined Daily Dosage of Antipsychotics (DDD-AP) assignment according to the World Health Organization was 1.08 (SD .61) [25]. Table 1 shows the demographics, diagnostic groups and symptoms of the 71 participants.

**Table 1:** Sociodemographic and clinical variables for 71 FEP patients

	**Mean**	**SD**
Age (years)	27.4	8.1

Education (years)	12.5	2.7

DUP (weeks) median/range	30	1–1040

GAF symptoms	42.8	14.0

GAF function	46.1	14.4

AES-C-Apathy	27.2	7.1

PANSS total score	60.5	15.1

PANSS positive	14.4	5.1

PANSS negative	14.7	5.8

PANSS general	31.3	7.4

CDSS	6.4	4.5

	**N**	**%**

Male gender	37	52%

Living alone	63	89%

Diagnosis		

Schizophrenia spectrum group	36	51%

Affective psychosis gorup	15	21%

Other psychosis group	20	28%

Abbreviations: DUP, duration of untreated psychosis; GAF, Global Assessment of Functioning Scale; PANSS, Positive and Negative Syndrome Scale; CDSS, Calgary Depression Scale for Schizophrenia; Schizophrenia spectrum group [schizophrenia (N = 30), schizophreniform (N = 6)]; Affective psychosis group [major depressive psychosis with mood incongruent psychosis (N = 12), Bipolar (N = 3)]; Other psychosis group [psychosis NOS (N = 13, brief psychosis (N = 6), delusional disorder (N = 1)]

### 2.2 Assessment

#### 2.2.1 Measures

#### Assessment of apathy

Apathy was assessed by the clinical version of the Apathy Evaluation scale (AES-C), an 18-item Likert scale ranging from 0–4 (0 = not at all and 4 = very much) [26]. The scale is based on Marin's definition of apathy as " diminished motivation and goal directed behavior, not attributed to diminished level of consciousness, general cognitive impairment or emotional distress" [26]. Examples of the questions are: "Are you interested in things?", "Is it important for you to get things done during the day?" and "Do you feel motivated?" The scale has been used across different medical disciplines. We have previously shown that a shortened 12-item AES-C scale (AES-C-Apathy) was a better assessor of apathy than the full version in a population with a FEP [27]. This abridged version was used in all the analyses in the present study.

#### Assessment of other symptoms and diagnosis

Symptoms were assessed by the Structural Clinical Interview of the PANSS (SCI-PANSS) [28]. Depression was assessed with the Calgary Depression Scale for schizophrenia (CDSS) [29]. Diagnostic assessment was carried out with the Structural Clinical Interview for DSM-IV (SCID-I interview) [30].

### 2.3 Neuropsychological assessments

A comprehensive neuropsychological test battery was administrated to all participants by psychologists or psychology students trained in clinical neuropsychology. The tests cover domains shown to be sensitive to the neurocognitive dysfunction of psychosis [31,32]: motor function (Grooved Pegboard) [33], psychomotor speed (Digit Symbol from WAIS-III) [34], attention (Digit Span forwards from WAIS-III) [34], working memory (Letter Number Span from WAIS-III) [34], verbal learning (California Verbal Learning Test; CVLT-II) [35], visual learning (Rey-Oesterrieth Complex Figure Test) [36] and executive function. For the executive function domain several tests from the Delis-Kaplan Executive Function System (D-KEFS) [37] were included in order to enable the investigation of the association of apathy with three different aspects of executive function; initiation, set shifting and inhibition. Initiation was assessed with Semantic fluency and Phonetic fluency (from the Verbal Fluency test) [37]. Set shifting was assessed with Category Switching (also from the Verbal Fluency test) [37], whereas inhibition was measured with the third trial on the Color-Word Interference test (the "Stroop" condition) [37]. Premorbid IQ was assessed with a Norwegian Research version of the National Adult Reading Test (NART) [38]; and current IQ with Wechsler Abbreviated Scale of Intelligence (WASI;) [39]. All participants showed adequate neuropsychological test effort indicated by two errors or less on the forced recognition trial of the CVLT-II. Table 2 gives the test results of the 71 patients.

**Table 2:** Neuropsychological test results for 71 FEP patients

Test	**Mean**	**SD**
*IQ*		

NART (N = 66)	14.4	7.3

WASI (N = 71)	105.2	13.7

*Motor function*		

Grooved Pegboard (N = 70)	110.2	26.5

*Psychomotor speed*		

Digit Symbol (N = 71)	63.5	14.4

*Attention*		

Digit Span forward (N = 71)	5.8	1.1

*Verbal memory*		

CVLT-II (N = 71)	53.0	10.5

*Visual memory*		

ROCF long term memory (N = 65)	19.5	6.7

*Working memory*		

Letter Number Span (N = 65)	9.7	2.5

*Executive function*		

Initiation		

Phonetic fluency (N = 71)	38.4	11.9

Semantic fluency (N = 71)	39.6	7.6

Set shifting		

Category switching (N = 71)	12.4	2.7

Inhibition		

Color-Word Interference (N = 71)	60.0	21.0

Abbreviations: NART, National Adult Reading test; WASI, Wechsler Abbreviated Scale of Intelligence; CVLT-II, California Verbal Learning Test II; ROCF, Rey-Osterrieth Complex Figure Test.

### 2.4 Procedures

All participants gave written informed consent to participate, and the study was approved by the Regional Committee for Medical Research Ethics and the Norwegian Data Inspectorate. The data file has received an Audit Certificate from the Center for Clinical research at Ullevål University Hospital.

The three investigators who did all the clinical assessments in the current study completed the common training and reliability program of the TOP study. Training in the AES-C was done by scoring videos, supervised by two experienced clinicians who had previously used the scale with other patient groups [40], and reliability testing of the AES-C was completed by seven live interviews with random study patients. The SCID training was based on the UCLA training program [41], and supervised by UCLA. For DSM-IV diagnostics, mean overall kappa for the standard diagnosis of training videos was 0.77, and mean overall kappa for a randomly drawn subset of actual study patients was also 0.77 (95% CI 0.60–0.94). Inter-rater reliability (Intra Class Coefficient (ICC) 1.1) for the different psychometric scales were: PANSS positive subscale 0.82 (95% CI 0.66–0.94) PANSS negative subscale 0.76 (95% CI 0.58–0.93), PANSS general subscale 0.73 (95% CI 0.54–0.90), GAF-S 0.86 (95% CI 0.77–0.92), GAF-F 0.85 (95% CI 0.76–0.92) and AES-C 0.98 (95% CI 0.92–0.99).

### 2.5 Analyses

#### 2.5.1 Data and statistical analyses

Analyses were performed with the statistical package SPSS, version 15.0 for Windows. A preliminary analysis was performed to examine the distribution of each variable. One patient was excluded because of being an extreme outlier on the Semantic fluency tests with 4 SD above the group mean (outlier score = 75; group mean = 40.1, SD = 8.6) influencing the results to such a degree that assumptions of homogeneity were violated. All tests were two-tailed, with a preset significance level of 0.05. Bonferroni corrections were applied in analyses where more than one test represented a neurocognitive domain, as noted in Table 3. Descriptive data are presented by either means or standard deviation (SD), or by median and range. Independent Student *t*-tests were used to analyze differences between groups. Associations between AES-C-Apathy and neuropsychological tests were investigated with Pearson product moment correlation analysis (r) and degree of influence by univariate regression analysis.

**Table 3:** Correlation between neuropsychological tests, AES-C-Apathy and possible confounding variables

Test (N)	**AES-C-Apathy**	**CDSS**	**PANSS Positive**	**DDD-AP**
	r	r	r	r

*IQ*				

Premorbid: NART	.11	<.01	.25*	.11

Current: WASI	-.15	-.02	-.11	-.23

*Motor function*				

Grooved Pegboard	.09	-.02	.14	.18

*Psychomotor speed*				

Digit symbol	-.17	-.04	-.11	-.15

*Attention*				

Digit span forwards	-.13	.02	.04	-.14

*Verbal memory*				

CVLT-II	-.15	.05	-.15	-.13

*Visual memory*				

ROCF long term memory	-.16	-.05	-.10	-.21

*Working memory*				

Letter Number Span	-.26*	-.07	-.13	-.13

*Executive function*				

Initiation				

Phonetic fluency^a^	-.25*	-.08	< -.01	-.17

Semantic fluency^a^	-.37**	-.05	-.04	-.11

Set shifting				

Category switching	-.18	.06	.02	-.24*

Inhibition				

Color-Word Interference	.23	.10	.25*	.10

r = Pearson product moment correlation; *p <.05; ^a ^Bonferroni corrected (0.05/2 = .03) * = p ≤ .03; ** = p ≤ .01

Abbreviations: NART, National Adult Reading Test; WASI, Wechsler Abbreviated Scale of Intelligence; CVLT-II, California Verbal Learning Test II; ROCF, Rey-Osterrieth Complex Figure Test; AES-C-Apathy, abridged Apathy Evaluation Scale; CDSS, Calgary Depression Scale for Schizophrenia; PANSS, Positive and Negative Syndrome Scale; DDD-AP, indicates defined daily dose according to the World Health Organization of Antipsychotic medication

Since apathy, negative symptoms and neurocognitive function have been found to be under influence from secondary sources, we took steps to control for this. Depression has been tested as a possible confounding variable for apathy in different studies in other medical disciplines [18,21], in addition positive symptoms and current use of antipsychotic medication has in some studies been found to influence either negative symptoms or neurocognitive function [42]. The association between these three variables and neurocognition were firstly examined in a correlation analysis followed by a hierarchical multiple regression analysis with the neurocognitive test results as the dependent. The possible confounders and AES-C-Apathy were the independent variables, with AES-C-Apathy entered in the last step.

## Results

### 3.1 The relationship between apathy, neuropsychological test performance and confounding variables

Three of the neuropsychological tests in the comprehensive TOP battery showed a statistically significant inverse association to AES-C-Apathy (Table 3). Out of these two (Semantic- and Phonetic fluency part of the Verbal Fluency tests) represented the initiation part of executive function, and the third (Letter Number Span) represented working memory. AES-C-Apathy was most strongly correlated with the Semantic fluency test (r = .37, p = .002), explaining 12% of its variance. For the two other tests (Phonetic fluency and Letter Number Span) the relationship to AES-C-Apathy was statistically significant, but of a smaller magnitude (r = .25, r = .26). AES-C-Apathy was neither significantly associated with any other tests, nor premorbid or current IQ.

There were no statistically significant differences in test performance for any neuropsychological tests (p value between .18 – .99) or in level of apathy (F = .16; p = .69) between the unmedicated patients (N = 14) and patients using antipsychotic medication (N = 57). None of the possible confounding variables (DDD-AP, PANSS positive symptoms or CDSS) had any significant correlations with the three neuropsychological tests that were statistically significantly associated with AES-C-Apathy (Table 3). This lack of influence was confirmed in a hierarchical regression analysis with Semantic-, Phonetic fluency and Letter Number Span as dependent variables. Here only AES-C-Apathy had a statistically significant contribution, even when entered in the last step (Table 4). The entry of the possible confounding variables did not change the influence AES-C-Apathy had on the neuropsychological tests in the univariate regression analysis (Table 4).

**Table 4:** Univariate and hierarchial multivariate regression analysis with neuropsychological tests as dependent and AES-C-Apathy and possible counfounders as independent variables

	**Dependent**				**Dependent**				**Dependent**			
	Letter Number Span				Phonetic fluency				Semantic fluency			

**Independent variables**	R^2^adj	β	t	p	R^2 ^adj	β	t	p	R^2 ^adj	β	t	p

**Univariate**												

AES-C-Apathy	.05	-.26	-2.2	**.04**	.05	-.25	-2.2	**.03**	.12	-.37	-3.3	**<.01**

**Hierarchial**												

DDD-AP	.01	-.13	-1.1	.30	.02	-.19	-1.6	.12	.00	-.12	-1.0	.31

PANSS positive	.01	-.06	-.5	.62	.01	.07	.6	.56	-.01	.04	.3	.75

CDSS	-.09	.05	.3	.72	-.02	.05	.4	.72	-.03	.15	1.2	.25

AES-C-Apathy	.04	-.27	-2.0	**.05**	.05	-.29	-2.2	**.03**	.12	-.44	-3.5	**<.01**

adj = adjusted; DDD-AP, indicates defined daily dose according to the World Health Organization of antipsychotic medication PANSS, Positive and Negative syndrome Scale; CDSS, Calcary Depression Scale for Schizophrenia; AES-C-Apathy, abridged Apathy Evaluation Scale

## Discussion

The main result of the present study is the replicated finding of a significant relationship between apathy and executive function in psychotic disorders, as found previously in one study of patients with chronic schizophrenia [9]. The two findings are corresponding even if the two studies applied different measures both for apathy and executive function, indicating a robust relationship. In addition, in both studies the relationship was not influenced by current level of depression. This supports the view that the presence of apathy and deficits in executive functioning are related in psychotic disorders, and that the relationship is not due to definitional issues since different scales and tests were used.

Additionally, this finding is in line with findings of an association between apathy and the specific executive functioning test of verbal fluency in several other brain disorders [18-20,22]. Two of these studies used the AES in assessing apathy [19,22], eliminating the challenge of comparing results between different scales.

This finding of a consistent relationship between apathy and tests of executive function across different brain disorders and across different levels of chronicity is supported by several studies that implicate prefrontal areas and frontal-subcortical circuitry involving the anterior cingular gyrus in both apathy and executive function [43-47].

In our opinion, our findings thus support the idea of a common or linked etiology between these negative symptoms and neurocognitive deficits as put forward in Harvey et al's model 2 and 3 [7]. This is in opposition to Harvey's own conclusions, that suggests that the two have different etiologies based on a lack of published studies showing significant relationships. In addition, our findings might imply that there are specific mechanisms behind the different negative subsymptoms. This is supported by a recent study finding that verbal memory was the only neuropsychological test to differentiate between those with and without flat affect, making the authors' suggest that this could reflect a unique neural substrate for this negative subsymptom [11].

We did not, as Roth et al, find associations between high levels of apathy and reduced IQ and memory, associations with apathy that are also found in other disorders. This could be due to the differences between the studies such as assessment of apathy, FEP patients being less chronically ill and diagnostically more diverse. But in order to understand more of the different negative subsymptoms, these associations should also be further studied.

The strength of our study is the use of a specific and validated assessment for the negative subsymptom of apathy together with a comprehensive neuropsychological test battery with tests for several different aspects of executive function. The main limitations to our study are: 1) that we only had access to instruments measuring one of the specific negative subsymptoms and thus cannot conclude anything about the specificity of our finding in regard to the other subsymptoms. 2) that for some patients the clinical assessment of apathy and the neuropsychological testing took place with a time difference and this could weaken our chances to detect weaker associations.

## Conclusion

First we replicated the finding in FEP patients, as was found in chronic patients, of a significant relationship between apathy and executive functioning. Second, we found that apathy in FEP have the same relationship to the Verbal Fluency test, as reported in other brain disorders, pointing to a common underlying nature of this symptom across disorders.

## Competing interests

The authors declare that they have no competing interests.

## Authors' contributions

AFa study design, collecting data, analysis, drafting and revising the manuscript. AV study design of neuropsychological tests, data collection (neuropsychological tests), data analysis and drafting and revising the manuscript. AFi study design, data analysis and drafting and revising the manuscript. IA conception of the study and revising the manuscript. EAB collecting data and revising the manuscript. SF study design, data analysis and revising the manuscript. CS data collection (neuropsychological testing) and revising the manuscript. OAA conception of the study and revising the manuscript. IM conception of the study, study design, data analysis and drafting and revising the manuscript.

All authors contributed to and have approved of the final version of the manuscript.

## Pre-publication history

The pre-publication history for this paper can be accessed here:


